# The building blocks of social communication

**DOI:** 10.2478/v10053-008-0145-6

**Published:** 2013-12-31

**Authors:** Margaret A. Niznikiewicz

**Affiliations:** Boston VA Healthcare Center, Harvard Medical School, Boston, MA, USA

**Keywords:** social cognition, ERP, social communication

## Abstract

In the present review, social communication will be discussed in the context of social cognition, and cold and hot cognition. The review presents research on prosody, processing of faces, multimodal processing of voice and face, and the impact of emotion on constructing semantic meaning. Since the focus of this mini review is on brain processes involved in these cognitive functions, the bulk of evidence presented will be from event related potential (ERP) studies as this methodology offers the best temporal resolution of cognitive events under study. The argument is made that social communication is accomplished via fast acting sensory processes and later, top down processes. Future directions both in terms of methodology and research questions are also discussed.

## Introduction

**Figure 1.**
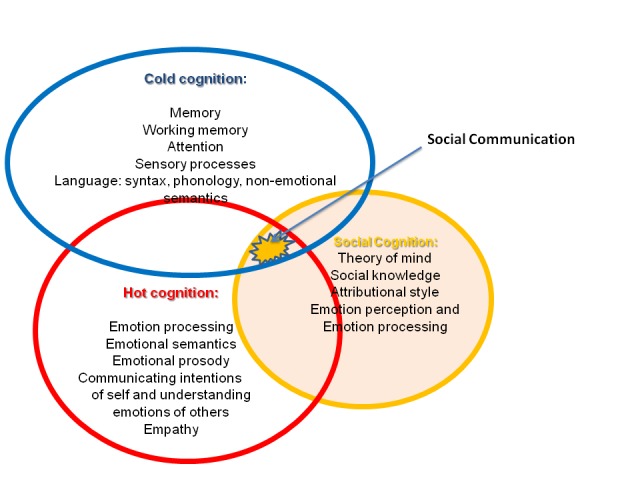
A model of interdependencies between “cold cognition,” “hot cognition,” and “social cognition.” The building blocks of social communication belong to all three domains.

After years of intense interest in different aspects of sensory and cognitive processes, more recently dubbed “cold cognition,” that included sensory analyses, attention, memory, language, and conflict resolution, to name a few, researchers realized that people are first and foremost social beings. Thus, one of the most important functions which these processes serve is social communication (Brück, Kreifelts, & Wildgruber, 2011; Decety & Svetlova, 2012). Neuroscience has contributed enormously to our understanding of how cold cognition processes are supported by the brain’s neural architecture and function (e.g., D’Ardenne et al., 2012; Newman, Carpenter, & Varma, 2003; Peelle & Davis, 2012; Poeppel, Emmorey, Hickok, & Pylkkänen, 2012). Likewise, technological advances and novel designs within the field of neuroscience allowed studying elements of social cognition at the brain level. They also significantly contributed to the broadening of a scope of inquiry into its constituents (Arlinger et al., 2009; Barraclough & Perrett, 2011). Accordingly, recent years have seen an enormous interestin the topic of *social cognition* defined as the ability to understand and interpret the intentions and emotions of others and adaptively react to these signals (Blakemore, 2012; Frith, 2012; Lieberman, 2012). In parallel to developments in research on social cognition, a line of research developed on the role of emotion and its interactions with other cognitive functions. Dubbed research on “hot cognition,” it examined different aspects of emotion processing. Like research on cold cognition and on social cognition, it has been enormously enriched by a neuroscience perspective. In Figure 1, I describe briefly, and non-exhaustively, research subsumed under the different rubrics of cold cognition, hot cognition, and social cognition, with an area “targeted” in this brief review marked with a star (see Figure 1). I will argue that in order to fully characterize human interactions with others, we should be focusing not only on the social cognition but, more broadly, on social communication. Social communication includes not only social cognition but also language and its interactions with emotion.

**Figure 2.**
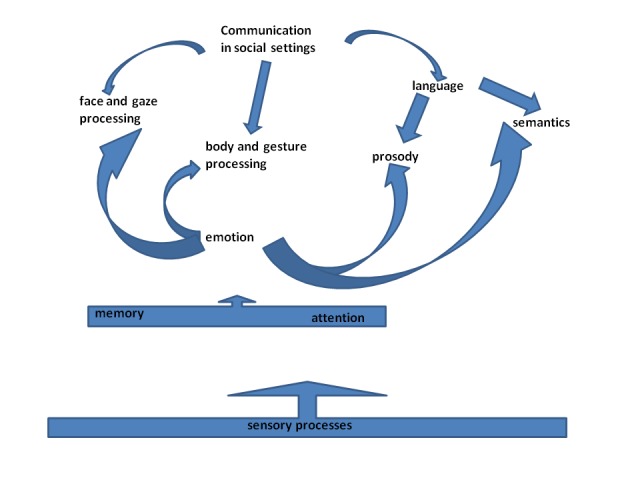
Social communication relies on language and its constituents, on gaze and facial expressions, as well as on body gestures. Sensory processes modulated by attention and memory processes provide raw data from which social meaning is constructed across all domains.

The building blocks of social communication are sensory processes that provide rapid analyses of incoming external stimuli, attentional processes that select sensory data for further processing as relevant for a task at hand, working memory processes that enable maintaining relevant information, and long-term memory structure and processes within it that allow rapid comparisons between oncoming information and existing semantic and semiotic knowledge (see Figures 1 and 2). A formal language system, both written and spoken, with the latter including syntactic and emotional prosody, is an important tool of social communication. It is only rivaled by facial and body gestures and expressions in conveying a full spectrum of human information, intentions, attitudes, and emotional states (see Figure 2). It is impossible to cover a topic of this complexity in a single paper.

Therefore, I will sample from different domains of research on social communication and focus on how neurophysiological processes contribute to its different instances.

As described above, the devices that people use to communicate with each other include their voice and its modulations: The voice modulations mark both syntactic structure of a sentence and emotional attitudes of a speaker (by prosody). They also include facial expressions and language. More recently, science has started to ask questions about brain processes involved in prosody and face processing as well as about how these two sources of affective information, voice and face, are processed together by the brain (see e.g., Ethofer et al., 2013; Jacob, Brück, Domin, Lotze, & Wildgruber, 2013). Another domain recently examined is the influence of affective states on cognitive processing, especially on language processing (e.g., Federmeier et al., 2001).

As indicated above, in this review, the emphasis will be on identifying neurophysiological processes that underpin these phenomena. Therefore, the methodology that I will focus on will be event related potentials (ERPs). ERPs remain the only existing technology that allows for capturing neurocognitive events with millisecond resolution as they unfold in real time. The overarching question in this enquiry into the different aspects of social communication is when the neurocognitive system distinguishes between different classes of sensory data such that, at a later stage, they become meaningful “building blocks” of social communication.

Accordingly, I will discuss studies examining prosody processing,including processing of non-verbal emotional vocalizations, face processing, simultaneous voice and face processing, and effects of emotion on semantic representations.

## Prosody processing

With a tone of voice we can express that we are happy, sad, angry, disappointed, or sarcastic. *Emotional prosody* refers to a tone of voice with which we speak. Prosodic information in a voice signal is primarily carried by fundamental frequency (F0) with contributions from other phonetic devices such as voice timbre, intensity, and speech rhythm (Schirmer & Kotz, 2006; Wildgruber, Ackermann, Kreifelts, & Ethofer, 2006). Recent functional studies established a network of brain regions that seem to be involved in prosody processing. They include superior and middle temporal gyrus (STG and MTG), parietal-temporal juncture, insula, as well as inferior frontal gyrus and orbitofrontal gyrus (Leitman et al., 2011; Schirmer & Kotz, 2006). ERP studies, on the other hand, pointed to the speed with which salient sensory features differentiating between different prosody types can be detected. Early prosody studies focused on exploring the question whether a neural system is sensitive to changes from one type of prosody to another. Most of these studies used a splicing technique that cuts an utterance into two different parts where the first one is said, for example, with a happy voice, and the second one with a sad voice (Paulmann & Kotz, 2008). This approach yields findings that point to the brain’s sensitivity to a shift of voice. Another class of ERP prosody studies focused on natural prosodic utterances (Kotz & Paulmann, 2007; Paulmann & Kotz, 2008; Pinheiro, Del Re, Mezin, et al., 2012). These studies point directly to the speed with which prosodic information is decoded and hint at the processing stages involved. In addition, they provide information on the functional architecture of the auditory system as applied to prosody processing.

All ERP studies of naturally occurring prosody report the N100 and P200 components of the waveform. The N100 is sensitive to sensory, physical properties of an acoustic signal, while the P200 has been associated with assigning sensory data different categorical values (Rosburg, Boutros, & Ford, 2008). Both components are believed to be modulated by attention. Since, by definition, the N100 peaks about 100 ms, and P200 about 200 ms after the onset of the stimulus, it appears that the first operations that allow assigning emotional valence to an auditory signal happen within the 200 ms from the onset of that signal. Most studies that focused on the analyses of the ERP correlates of natural prosody processing formally examined only the P200 component. Most of them indicated that, at the level of the P200, the prosodic signal is categorized into emotional and non-emotional rather than into discrete emotional categories such as happy, angry, or sad (Kotz & Paulmann, 2007; Paulmann & Kotz, 2008; but see Pinheiro, Del Re, Mezin, et al. 2012, where the distinction was found in the P200 amplitude between neutral relative to happy, relative to angry prosody).

**Figure 3.**
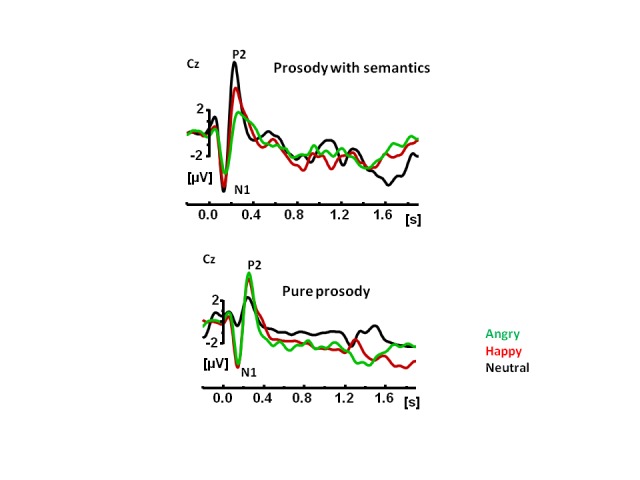
Grand average waveforms timelocked to the onset of a sentence to sentences with prosodic and semantic content and to pure pro- sody sentences in 15 individuals. The prosody types were neutral, happy, and angry. Adopted from “Sensory-Based and Higher-Order Operations Contribute to Abnormal Emotional Prosody Processing in Schizophrenia: An Electrophysiological Investigation,” by A. P. Pinheiro, E. Del Re, J. Mezin, P. G. Nestor, A. Rauber, R. W. McCarley, et al., 2012, *Psychological Medicine*, 43, 603-618.^1^

The Pinheiro, Del Re, Mezin, et al. study (2012) was one of the first to formally analyze the N100 in terms of its sensitivity to prosodic information. We used simple neutral sentences that either carried semantic information intact or removed by an acoustic manipulation. In semantically intact sentences, the N100 was more negative to neutral prosody relative to angry prosody (see Figure 3). For the same stimuli, when they were stripped off their semantic content, the N100 was more negative to the neutral relative to the emotional prosody (see Figure 3). These results lead to the conclusion that emotional meaning is constructed from the sensory data whose physical properties are processed differentially from the early stages of analyses. In a series of analytic steps, they are endowed, or tagged, with increasingly rich “meaning.” These data also suggest that physical properties of a signal matter, such as in a differential N100 response to intelligible versus non-intelligible speech. Since in normal populations the size of the N100 is associated with the ease of processing a given stimulus, we can further speculate that the physical properties of neutral undistorted speech are easier to process than the same speech when it is distorted. Interestingly, the pattern for the emotional prosody for the distorted and undistorted speech was opposite to that observed for the neutral prosody. It remains an issue of further research to examine whether these differences have to do solely with the physical properties of the speech signal or whether they are mediated via attentional and, perhaps, memory mechanisms.

The P200 in this study was also sensitive to prosodic information similarly to previous ERP prosody studies. Similarly to the N100 results, the pattern of amplitude differences was different in sentences with and without semantic content. Interestingly, unlike in earlier studies, the P200 amplitude did distinguish between different types of prosody: neutral, happy, and angry (see Figure 3).

**Figure 4.**
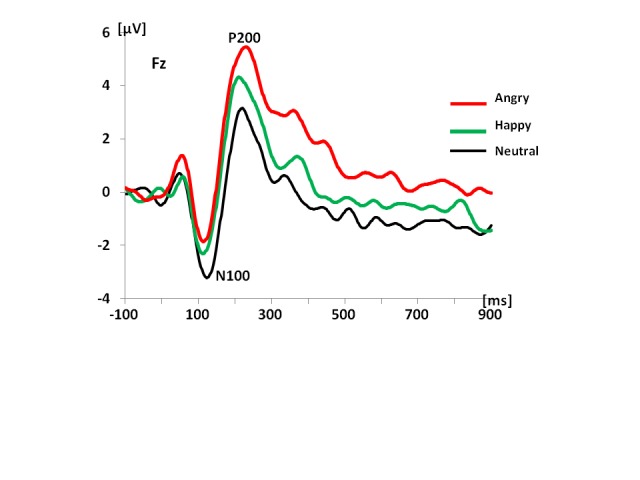
Grand average waveforms to non-semantic vocalizations: neutral, angry, and happy in 19 individuals. Adopted from “Electrophysiological Insights Into Processing Non-Verbal Emotional Vocalizations,” by T. Liu, A. P. Pinheiro, G. Deng, P. G. Nestor, R.W. McCarley, and M. A. Niznikiewicz, 2012, *NeuroReport*, 23, p. 108-112 (see Footnote 1).

The speed with which prosodic information is processed and a role of sensory and early categorization processes were further explored in a study of non-verbal human sounds that connoted neutral, happy, and angry emotional states (Liu, Pinheiro, Deng, et al., 2012). In that study, we used neutral “mm” sounds, laughs, and angry sounds to examine whether such non-semantic emotional sounds will be associated with similar brain responses to those observed for sentences. There were notable similarities and differences between the ERP results recorded to non-semantic emotional vocalizations relative to emotional prosody in sentences. As in the study of prosody processing in sentences, both N100 and P200 were sensitive to different emotional voices (see Figure 4). However, for these non-semantic sounds, the first differentiation between different vocal emotions was observed at the level of P50 which was more positive to angry sounds relative to either happy or neutral vocalizations (see Figure 4). One can only speculate that perhaps angry sounds have the most ecologically valid salience relative to the two otheremotions: happy and neutral. The N100 effects were similar to those observed in the sentence prosody study in that the N100 was more negative to neutral relative to emotional vocalizations. However, the P200 effects were similar to those observed in the sentence prosody study for distorted (i.e., non-semantic) sentences in that the P200 was more positive to emotional relative to neutral vocalizations (see Figure 4).

Together, these results suggest that vocal emotion, whether it is a patterned prosody found in sentences or emotional non-semantic vocalizations, is processed by the brain fairly rapidly within the first 200 ms after a stimulus onset. However, the specific processes as indexed by the N100 and P200, and possibly by earlier components, will depend on the particular sensory properties of an acoustic signal, and perhaps, on interactions of these sensory processes with modulations from attentional and memory processes.

## Face Processing

The face is another important source of social information. Even without using words we can express a whole range of emotions ranging from neutral expressions through boredom, happiness, to fear, and anger. Functional imaging studies point to fusiform gyrus, and especially fusiform face area as well as occipital face area as involved in processing information from the face (e.g., Rhodes, Byatt, Michie, & Puce, 2004) with contributions from inferior frontal gyrus, amygdala, and superior temporal sulcus (Cohen Kadosh, Cohen Kadosh, Dick, & Johnson, 2011; Cohen Kadosh, Johnson, Henson, Dick, & Blakemore, 2012; Skelly & Decety, 2012). It takes the first decade of a person’s life to develop efficient strategies to process faces.

The first reports of an ERP response specific to faces came from McCarthy and Puce who identified N170 (Allison, Puce, Spencer, & McCarthy, 1999; Puce, Allison, & McCarthy, 1999) as the potential sensitive to face processing, especially structural face processing (McCarthy, Puce, Belger, & Allison, 1999), with numerous studiesfollowing and examining different aspects of face analysis (for a review, see Boehm & Paller, 2006). The N250 was also reported to be sensitive to emotional face processing (for a review, see Eimer & Holmes, 2007).

As with prosody processing, it is noteworthy that the first differentiation of sensory data related to face falls within the first 200 ms from the onset of a stimulus.

## Multimodal processing of face and voice

As reviewed above, the first wave of studies on the brain correlates of emotion processing focused separately on the processing of voice and of face information. However, in real life, voice and face information is rarely processed separately and more recent studies addressed the issue of simultaneous processing of socially relevant information from these two modalities. The questions addressed by a handful of studies on multimodal face and voice processing focused on two major issues: (a) Which brain regions/networks uniquely support multimodal face and voice processing (functional magnetic resonance imaging [fMRI] studies), and (b) what cognitive processes are associated with multimodal face and voice processing. Thus far, most studies have been conducted using fMRI methodology (e.g., Klasen et al., 2012). In spite of the fact that many details of the brain architecture involved in different aspects of multimodal face and voice processing are missing, the network of main regions involved has been delineated. Kreifelts et al.’s (2007) fMRI study of several emotions (neutral emotion included) presented in auditory, visual, and audiovisual modalities identified posterior superior temporal gyrus (pSTG) and right thalamus as regions which showed more activation in the audiovisual relative to auditory or visual stimuli. In addition, better accuracy of emotion identification was observed for audiovisual stimuli. In the follow-up study using the same type of stimuli, Kreitfelts et al. (2009) identified different parts of the superior temporal sulcus (STS) as more sensitive to information from voice and face; in that study, the area sensitive to audiovisual information was at the interface between the regions mostly sensitive to voice or to face. Joassin et al. (2011) examined cross modal interactions associated with the process of a person’s identity recognition. Using voices, static faces, and face plus voice stimuli, the authors identified both unimodal regions supporting voice and face processing, and multimodal regions including the left angular gyrus and the right hippocampus as sensitive to the processing of face-voice pairings.

The two ERP studies examining temporal dynamics of information from face and voice (Jessen & Kotz, 2011; Latinus, VanRullen, & Taylor, 2010) emphasized early sensitivity to multimodal information. In the Latinus et al. study, the early effects were found within 30-100 msand at later stages around 180-320 ms, post-stimulus. In the Jessen and Kotz study, the effects were found on the N100 that was reduced to audiovisual stimuli relative to auditory stimuli.

In our inquiry into how multimodal emotional cues are processed by the brain (Liu, Pinheiro, Zhao, et al., 2012), we presented neutral, happy, and angry faces whose presentation was time-locked to the onset of neutral, happy, or angry vocalizations. A complex set of components was recorded in the audiovisual condition that was different both from a pattern of components recorded in either auditory or visual modality (see Figure 5). We observed two distinct patterns of ERP components: parietally distributed N100, N170, and P270, and fronto-centrally distributed N100, P200, N250, and P300. Of note, the parietal components, even though they were clearly sensitive to face stimuli, did not distinguish between different emotional states. Only fronto-central components showed differential sensitivity to emotional states. The components that clearly distinguished between neutral and emotional face/voice pairings were P200, N250, and P300 associated with initial categorization processes, assigning emotional valence to stimuli, and attentional processes, respectively (see Figure 5). Overall, these results suggest that a processing stream takes actually longer and is more complex for face-voice stimuli than for either voice (indexed with N100 and P200) or face alone (indexed with N170 and in some designs with P300).

**Figure 5.**
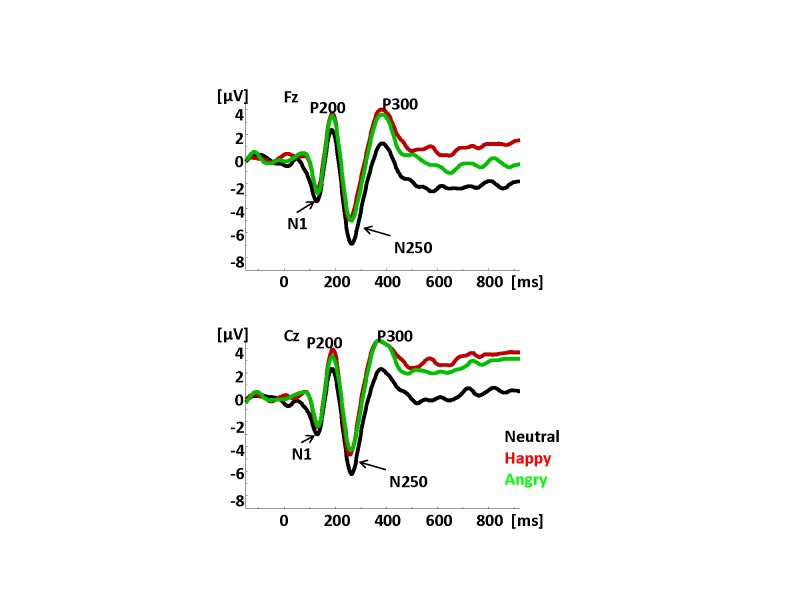
Grand average waveforms to face and voice (multimodal) stimuli, to face only, and to voice only stimuli in 18 individuals. Panel A. Fronto-central components sensitive to the distinction between neutral and emotional face/voice pairings. Adopted from “Emotional Cues During Simultaneous Face and Voice Processing: Electrophysiological Insights,” by T. Liu, A. P. Pinheiro, Z. Zhao, P. G. Nestor, R. W. McCarley, and M. A. Niznikiewicz, 2012, *PLOS ONE*, 7(2), e31001 (see Footnote 1).

**Figure 5 (continued).**
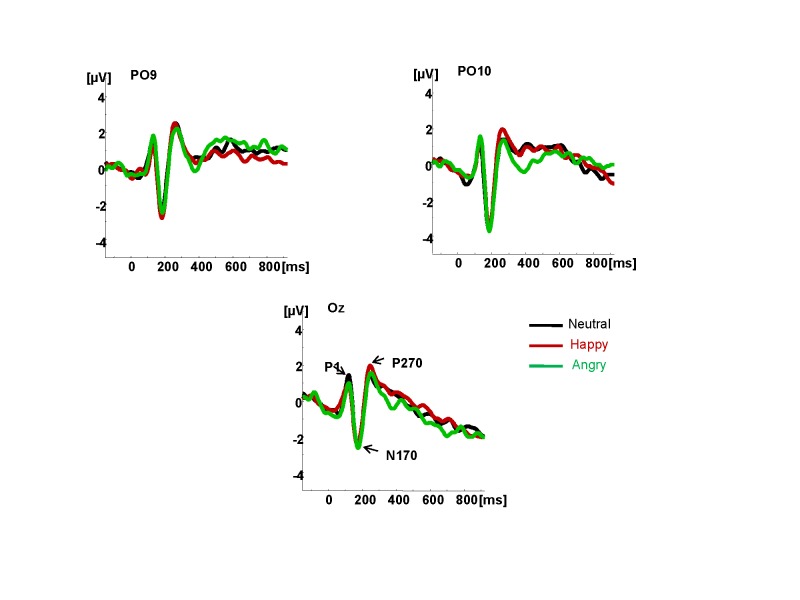
Grand average waveforms to face and voice (multimodal) stimuli, to face only, and to voice only stimuli in 18 individuals. Panel B. Parietal-occipital components indexing structural face processing. Adopted from “Emotional Cues During Simultaneous Face and Voice Processing: Electrophysiological Insights,” by T. Liu, A. P. Pinheiro, Z. Zhao, P. G. Nestor, R. W. McCarley, and M. A. Niznikiewicz, 2012, *PLOS ONE*, 7(2), e31001 (see Footnote 1).

## The interactions between mood and semantic memory contents

The component that is sensitive to semantic incongruity is the N400 that peaks around 400 ms after a target word was presented (Deacon, Hewitt, Yang, & Nagata, 2000; Holcomb, 1993; Kutas & Federmeier, 2000, 2011). The N400 is least negative if a word fits into the preceding context well, and is significantly more negative if it does not (Kutas & Federmeier, 2011). The N400 has been used successfully in numerous studies of language to probe processes and structure of semantic memory, as well as the way in which meaning is constructed by the human brain (e.g., Federmeier & Kutas, 1999a, 1999b; Kutas & Federmeier, 2011).

More recently, it has been demonstrated that the N400 can be a useful probe of the way in which emotional states influence the processing of meaning. Behavioral studies suggest that positive mood helps generate more associations and results in greater cognitive flexibility, but also contributes to making more errors. In contrast, negative mood narrows a range of associations but is also related to making fewer errors (e.g., Bolte, Goschke, & Kuhl, 2003; Dreisbach, 2006; Dreisbach & Goschke, 2004; Fiedler, 1988, 2001; Isen & Daubman, 1984; Isen, Niedenthal, & Cantor, 1992). However, behavioral data do not allow inferences about neurophysiological processes that underpin these effects.

A handful of ERP studies examined an interaction between semantics and affect from several related angles. Overall, their results strongly suggest interactions between semantics and emotion with specific studies providing evidence for different facets of these interactions. Goerlich et al. (2012) demonstrated that meaning carried by single words is processed faster and is associated with a more reduced N400 if the target word is preceded by an emotional stimulus congruent with the valence of the target word. An interaction between information structure and emotion was examined in the Wang, Bastiaansen, Yang, and Hagoort (2013) study. In that study, an interaction was found between emotionally salient words and information structure at the level of the N400: Semantic integration was influenced by information structure only for neutral words but not for emotional words. It was suggested that the greater emotional salience of emotional words overrode the influence of information structure. Chwilla, Virgillito, and Vissers (2011) took a slightly different approach by examining an interaction between N400 and mood where subjects’ mood was manipulated with a mood induction procedure. The study was designed to test two competing theories of language: Embodied theories of language use suggest that symbols used in language are grounded in perception, action, and emotion, while abstract symbol theories suggest that meaning is constructed from syntactic combinations of abstract symbols (Chwilla et al., 2011). Within this context, interactions between semantics and emotion are treated as support for the embodied theories of language use. In this study, the N400 was differentially affected by induced mood lending support for the embodied theories of language.

Even though the Federmeier, Kirson, Moreno, and Kutas (2001) study can be interpreted within the same framework as the Chwilla et al. (2011) study, the authors emphasized interactions between the contents and structure of semantic memory and emotional states of language users. They tested directly the premise that positive mood broadens a pool of semantic associations and thus makes it easier to accept words that fit context less well. The authors used a series of two-sentence mini-paragraphs in which the last word in a second sentence was manipulated such that it either formed a well-form ending (expected ending), an ending that was incongruent with a sentence previous context but belonged to the same category as the correct sentence ending (within category violation), or was an incorrect sentence ending from a different semantic category (between category violation). For example, expected ending: “Paul loved to watch all TV shows that were set in hospitals. He has always wanted to be a *doctor*”; within category violation: “Louise was suffering from a toothache for several days, but she still refused to do anything about it. She has always been afraid of going to the *doctor*”; between category violation: “Michaela loved to read and she couldn’t wait to check out a new set of books. She knew they were set aside for her by the friendly *doctor*” (examples from Pinheiro, Del Re, Nestor, et al., 2012). Accordingly, under positive mood, the N400 to within category violations (i.e., to words that did not fit the context well but belonged to the same category as the targets) was not different from the N400 to expected endings. This result confirmed that positive mood indeed broadens a pool of semantic associations such that words poorly fitting the context but associated with a good fit are treated by the cognitive system as acceptable - as indexed by a reduced N400.

**Figure 6.**
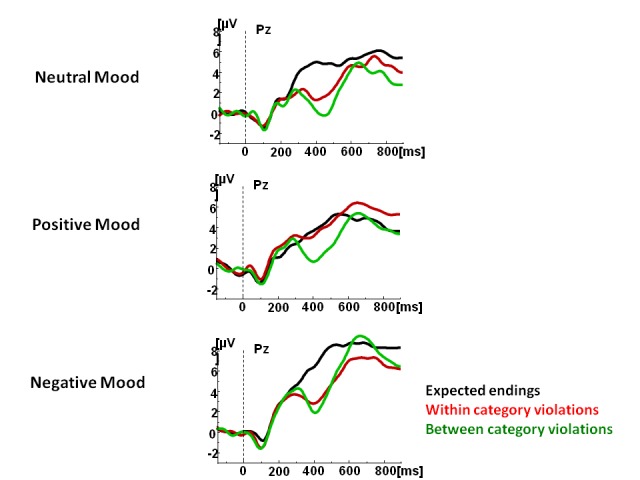
Grand average waveforms to final words in the two-sentence paragraphs that constituted expected endings, within category or between category violations in 15 individuals. Adopted from “Interactions Between Mood and the Structure of Semantic Memory: Event-Related Potentials Evidence,” by A. P. Pinheiro, E. Del Re, P. G. Nestor, R. W. McCarley, O. F. Gonçalves, and M. A. Niznikiewicz, 2012, *Social Cognitive and Affective Neuroscience*, 8, 579-594 (see Footnote 1).

We have replicated and extended this finding by testing the effects of both positive and negative moods using a new set of stimuli (Pinheiro, Del Re, Nestor, et al., 2012). We used the two-sentence paragraph paradigm described above and presented it to participants under three different moods: neutral, positive, and negative, with the order of presentation counter-balanced across participants. The N400 response to within category violations changed in the mood-specific manner. Under the positive mood, the N400 to within category violations became not significantly different from the N400 to correct targets suggesting that under the positive mood, within category violations were accepted as correct sentence endings (see Figure 6). Conversely, under the negative mood, the prediction mechanisms were altered such that the within category violations were treated similarly to the between category violations, that is, items that shared few semantic features with the target word (see Figure 6). Thus, it appears that mood modulates the interactions between contents of semantic memory and meaning construction. It is not entirely clear how such influence might be exerted but speculations include a role of several neurotransmitters including serotonin, norepinephrin, and dopamine that play a role both in emotion regulation and in the context use (Ruhe, Mason, & Schene, 2007).

## Summary

This quite limited overview of research on social communication that includes different aspects of emotion processing from voice and from voice and face, as well as the influence of emotion on a cognitive processing style, exemplifies well how these behaviors are instantiated in the brain. First, it is apparent that the neurophysiological processes involved are relatively fast and span from 50 ms to 450 ms after the stimulus onset. Second, emotional information from voice and face is first tagged and segregated into processing streams at the level of physical features that have ultimately emotional valence assigned. For example, while similar ERP components, and thus, presumably, similar neurophysiological events were associated with the processing of semantic and non-semantic prosody, there were also differences between these two types of processes. The non-semantic affect seems to be processed into neutral and emotional types without the distinction of the type of semantic emotion, while semantic prosody seems to be segregated according to emotion type. As one might expect, the joint processing of face and voice is associated with a more complex set of the ERP components, and thus more complex processes, than processing of the face or the voice alone.

In terms of both methodological and theoretical considerations, it is important to keep in mind that the ERP effects observed between 50 to 300 ms for voice, and voice and face processing, do not represent all processes that are involved in affect processing from voices and faces. For example, it has been demonstrated that orbito-frontal gyrus (OFG) is involved in assigning emotional valence to a voice signal, and that ERPs are not sensitive to activity found in the orbito-frontal gyrus (Paulmann, Seifert, & Kotz, 2009). The activity in the OFG comes relatively late in the processing stream. Similarly, it is apparent that emotion is processed both on cortical and sub-cortical levels and at least some of the subcortical processes are not reflected in the surface ERPs. Finally, as is evident from the work of LeDoux and others, emotion networks, including cortical and sub-cortical regions, are both complex and emotion specific (LeDoux, 2000; Johansen, Wolff, Lüthi, & LeDoux, 2012). The full account of emotional cues processing from both voice and face will have to take into consideration evidence from multiple methodologies and experimental approaches.

The interaction between mood and cognitive processing style is even more complicated. Mood elicitation is associated with a unique set of processes not discussed here. Their result is a shift in mood that, at the brain level, is likely associated with a differential set of specific brain regions’ sensitivities to signals mediated by changes in neurotransmitter levels. These brain changes result in cascade reactions where the same semantic information is processed somewhat differently de-pending on an emotional framework provided by the mood.

This line of research demonstrates how elements of social cognition emerge from sensory data and hints at interactions with higher order processes. Some of the findings in this domain have been highlighted. However, even from this limited review, it is apparent that our understanding of both sensory processes that operate on physical data, and of higher order cognitive processes that modulate them, is still quite limited. It is even less understood what is the nature of the interactions between sensory and higher cognitive processes that ultimately leads to a rich experience of socially relevant events.

In terms of methodology, this review points to the limitations of just one imaging methodology, here ERPs. It is suggested that multimodal assessment of these complex behaviors will bring us closest to their understanding. One technique that is gaining popularity is multimodal fusion of brain imaging data where information from several (at least two) imaging methodologies is used to describe cognitive phenomena under study (Sui, Adali, Yu, Chen, & Calhoun, 2012). It is hoped that the use of multimodal data fusion techniques will not only enhance the sophistication of the tools used to ask important questions but will also contribute to a deeper understanding of how an act of social communication is accomplished across brain regions and brain systems.

Finally, several aspects of social communication are still not very well understood. To name a few, the function of body language and its brain correlates is not well delineated. With the exception of a few studies, relative contributions of eye gaze and facial expressions to convey socially relevant information are also not well described. While not discussed in this review, the role of a theory of mind and empathy in shaping social communication and brain processes that support these processes are also just beginning to be unraveled.

While these questions present challenges, they also present exciting opportunities to better understand the human brain as an organ of social cognition. In so doing we may be able to help these individuals for whom effective social communication is difficult to achieve.

## Footnotes

1 While the figures bear resemblance to already published figures, they were generated from our own data and no part of the published figures was used in their creation.
